# Weighted gene co-expression network analysis identified MYL9 and CNN1 are associated with recurrence in colorectal cancer

**DOI:** 10.7150/jca.39723

**Published:** 2020-02-10

**Authors:** Xiao Qiu, Shen-Hong Cheng, Fei Xu, Jin-Wen Yin, Li-Yang Wang, Xin-You Zhang

**Affiliations:** 1 Department of Hematology, Shenzhen People's Hospital, The Second Clinical Medical College of Jinan University, The First Affiliated Hospital of Southern University of Science and Technology, Shenzhen, China; 2 College of Basic Medicine, Army Military Medical University, Chongqing, China; 3 Department of Gastroenterology, Zhongnan Hospital of Wuhan University, Wuhan, China; 4 Department of Gastroenterology, Shenzhen People's Hospital, The Second Clinical Medical College of Jinan University, The First Affiliated Hospital of Southern University of Science and Technology, Shenzhen, China

**Keywords:** colorectal cancer (CRC), weighted gene co-expression network analysis (WGCNA), recurrence, hub gene

## Abstract

Colorectal cancer (CRC) is one of the most common carcinomas and the fourth leading cause of cancer-related death worldwide. One of the obstacles in the successful treatment of CRC is a high rate of recurrence. We aimed to construct weighted gene co-expression network analysis (WGCNA) to identify key modules and hub genes in association with recurrence in CRC patients. We firstly used the microarray data, GSE41258, to construct a co-expression network and identify gene modules. Furthermore, protein and protein interaction (PPI) network was also performed to screen hub genes. To validate the hub genes, an independent dataset GSE17536 was used for survival analyses. Additionally, another two databases were also performed to investigate the survival rates and expression levels of hub genes. Gene set enrichment analyses (GSEA) combined with gene ontology (GO) were performed to further explore function and mechanisms. In our study, the midnightblue module was identified to be significant, 15 hub genes were screened, four of which were identified as hub nodes in the PPI network. In the test dataset, we found higher expression of MYL9 and CNN1 were significantly associated with shorter survival time of CRC patients. GO analyses showed that MYL9 and CNN1 were enriched in “muscle system process” and “cytoskeletal protein binding”. GSEA found the two hub genes were enriched in “pathways in cancer” and “calcium signaling pathway”. In conclusion, our study demonstrated that MYL9 and CNN1 were hub genes associated with the recurrence of CRC, which may contribute to the improvement of recurrence-free survival time of CRC patients.

## Introduction

Human colorectal cancer (CRC) is a global cause of concern in terms of both morbidity and mortality. It is recognized as the third most common carcinoma and the fourth leading cause of cancer-related death in the world 1. Although tremendous advances have been made for diagnosis and treatment of CRC, the burden of disease is still high 2. One of the deficiencies in the completely cure of CRC is the poor prognosis, such as a high rate of tumor recurrence. It is estimated that 30% of patients with stage I-III and 65% of patients with stage IV CRC suffer recurrence after curative treatment 3. No matter whether patients have received a series of progressive adjuvant therapies, however, early detection of disease recurrence during follow-up period results in improved long-term outcomes in CRC patients 4-6. Therefore, knowledge of prognostic and predictive biomarkers for recurrence may effectively contribute to better guidance to selection of treatment strategy as well as improved prognosis.

With the discovery and progression of microarray technologies with high throughput, it is the fact that gene expression profiles have been widely applied in the research area of cancer. Most microarray analyses have attached value to the comparison between tumor and normal samples 7. With the growing interest in bioinformatics analysis, weighted gene co-expression network analysis (WGCNA) has emerged as a novel approach to perform holistic microarray analyses, which is able to identify not only differentially expressed genes (DEGs), but also high degree of interconnection between genes 8. In WGCNA, the basic concept is construction of co-expression modules, which are clusters of genes maintaining consistent expression patterns and even playing similar biological roles, and these modules are derived from the data of mRNA expression profiles by performing unsupervised hierarchical clustering 9-11. In recent years, WGCNA has been successfully applied in the investigation of tumors, such as CRC. It has been reported that four key lncRNAs (RP11‐33O4.1, PCGF5, RP11‐164P12.5 and CTD‐2396E7.11) were found to participate in the development of colon adenocarcinoma (COAD) 12. In addition, COL8A1 was demonstrated to be related to prognosis and progression of COAD by constructing WGCNA as well 13. With the exploration of a growing number of mRNA expression files of CRC in different databases, we get opportunities to analyze the mRNA expression data of genes derived from a variety of platforms and institutes 14. Exploring more different potential biomarkers for different clinicopathological variables of CRC is of great significance.

In our study, for purpose of improving the knowledge of biological mechanisms underlying recurrence of CRC, WGCNA was applied to identify hub genes associated with the recurrence of patients with CRC.

## Materials and Methods

### Data Collection

The mRNA expression profiles of human CRC with contained clinical information of patients were downloaded from GEO online database. GSE41258 and GSE17536 were two datasets performed on Affymetrix Human Genome U133A Array (HG-U133A). GSE41258 was used as a training set by constructing a co-expression network then identifying hub genes, which included 182 CRC samples and 54 normal ones 15. In addition, GSE17536 was used as an independent test dataset, including 177 CRC patients 16.

### Data Preprocessing

The “affy” package was used to preprocess and normalize the raw microarray data 17. Microarray quality was evaluated by the methods of intuitive observation, averaging and data fitting 18. First, we could obtain a grey-scale map by using the function of “image” in order to get a view of the signal intensity of microarray on the whole. Then, the “simpleaffy” package was conducted to get an overview diagram of quality assessment 19, 20. Furthermore, data fitting was conducted by the “affyPLM” package. Finally, sample clustering was conducted to detect microarray outliers on the basis of distances between samples in Pearson's correlation matrices and average linkage. The top 5000 varying genes were chosen for WGCNA.

### Construction of WGCNA

R package “WGCNA” was conducted. First, an appropriate soft thresholding power was determined by the approximate scale-free topology criterion 21, 22. After that, the adjacency was transformed into topological overlap matrix (TOM), followed by calculation of corresponding dissimilarity (1-TOM) 23. Then, identification of modules was accomplished through the method of dynamic tree cut. It was usually determined by hierarchically clustering genes with similar expression profile and using 1-TOM dissimilarity with a minimum size of 30 cut-off 24. Finally, we selected a cutline for module dendrogram to merge modules for further analysis by calculating the dissimilarity of module eigengenes (MEs).

### Identification of Clinically Significant Modules

ME and module significance (MS), two important parameters, were used to identify modules related to clinical traits of CRC patients. First, ME was the major component of a particular gene module, correlation between clinical features and MEs were calculated. Second, MS was regarded as the average value of gene significance (GS) in a particular module. Hence, the highest absolute MS was usually thought to be a persuasive indicator when selecting a clinically significant module.

### Identification of Hub Genes

Hub genes were measured by the clinical feature relationship (cor.geneTraitSignificance > 0.2) and the absolute value of the Pearson's correlation (cor.geneModuleMembership > 0.8), which showed a high network connectivity in a particular module 25, 26. Additionally, by uploading all genes in the clinically significant module of interest to the Search Tool for the Retrieval of Interacting Genes (STRING) database (https://string-db.org/) 27, protein and protein interaction (PPI) network was constructed with confidence > 0.4. In the PPI network, hub nodes were genes with connectivity degree ≥ 4 (node/edge). Collectively, genes in both co-expression network and PPI network were selected as candidate hub genes for further analysis. The DEGs with |log2fold change (FC)| > 1 and false discovery rate (FDR) < 0.05 were statistically significant in GSE41258 28.

### Validation of Hub Genes

First, a test dataset, GSE17536, was used for survival analyses of above candidate hub genes. In the dataset, 177 samples were divided into low and high groups according to the medium value of the expression of genes. Then “survival” package in R was performed. The log-rank test was adopted to compare two groups 29. Furthermore, Gene Expression Profiling Interactive Analysis (GEPIA) (http://gepia.cancer-pku.cn/) database was used to validate outcomes of survival analyses and gene expression levels 30. Moreover, the Human Protein Atlas (https://www.proteinatlas.org/) database was used for immunohistochemistry (IHC) analysis.

### Function Enrichment Analysis

To get further insight into the function of hub genes in the module of interest, we performed Gene Ontology (GO) enrichment analysis by loading "anRichment" package in R. P < 0.05 was set as the cut-off criterion.

### Gene Set Enrichment Analysis (GSEA)

In the test dataset GSE17536, 177 samples with CRC were grouped into low and high groups, according to the median value of the expression levels of genes. To explore mechanisms of hub genes in CRC, GSEA 31 was performed and mapped into Kyoto Encyclopedia of Genes and Genomes (KEGG) pathway enrichment database. c2.cp.kegg.v5.2.symbols.gmt was selected in this study, which was used as reference gene sets. FDR < 0.05 was chosen as the cut-off criteria.

### Collection of clinical tissue samples

30 paired CRC tissues and adjacent tissues were collected from the Department of General Surgery, Zhongnan Hospital of Wuhan University (Wuhan, China). The study was approved by the ethics committee of Zhongnan Hospital of Wuhan University, in accordance with the Declaration of Helsinki. Written informed consent was obtained from the enrolled patients. The samples were directly dissected and subjected to RNA extraction.

### Quantitative Real-Time PCR (qRT-PCR)

Total RNA was extracted from 30 paired tissues with TRIzol reagent (Invitrogen, USA). Total RNA (1 μg) was used to synthesize first-strand cDNA using a synthesis kit (Thermo Fisher Scientific, USA). The experiments were performed by using a QuantStudioTM 6 Flex Real-Time PCR instrument (ABI, USA) with SYBR® Premix Ex TaqTM II Mix (Takara, Japan). GAPDH was taken as the internal control. The relative mRNA expression levels were calculated using the 2^-ΔΔCt^ method 32. The gene specific primers were as follows: MYL9 (Forward 5'-GGATGTGATTCGCAACGCCTTTG-3' and Reverse 5'-CGGTACATCTCGTCCACTTCCT-3'); CNN1 (Forward 5'-CCAACGACCTGTTTGAGAACACC-3' and Reverse 5'-ATTTCCGCTCCTGCTTCTCTGC-3'); GAPDH (Forward 5'-AGAAGGCTGGGGCTCATTTG-3' and Reverse 5'-GCAGGAGGCATTGCTGATGAT-3').

## Results

### Data Preprocessing

**Figure 1:**
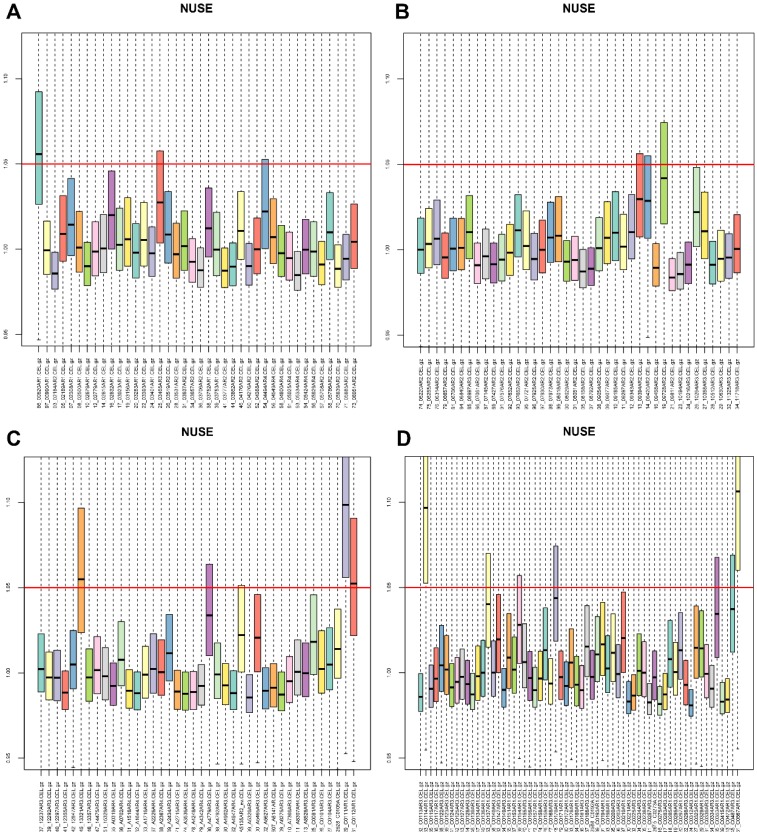
The normalized unscaled standard errors (NUSE) of 182 CRC samples in GSE41258. CRC, colorectal cancer.

First, we performed an overview diagram of quality assessment of total 182 CRC samples (Fig. S1). It is recognized that the absolute value of GAPDH 3'/5' should not be greater than 1 20. Fig. S1c showed that GSM1012445 was unqualified. Furthermore, GSM1012445 was chosen for data fitting analysis. Weights plot, residuals plot, relative log expression (RLE) boxplot and normalized unscaled standard errors (NUSE) boxplot were shown (Fig. S2, S3 and Fig. 1). NUSE is more sensitive than RLE, it is suggested that the samples were unqualified when the NUSE deviated from 1. Therefore, our results showed that GSM1012286, GSM1012445, GSM1012529, GSM1012531, GSM1012533 and GSM1012651 (Fig. 1a, c, d) were unqualified. Moreover, another three samples (GSM1012525, GSM1012326 and GSM1012604) were also removed by performing sample clustering (Fig. S4). To sum up, a total of nine samples should be removed from subsequent analyses in GES41258.

### Weighted Co-expression Network Construction and Key Modules Identification

**Figure 2:**
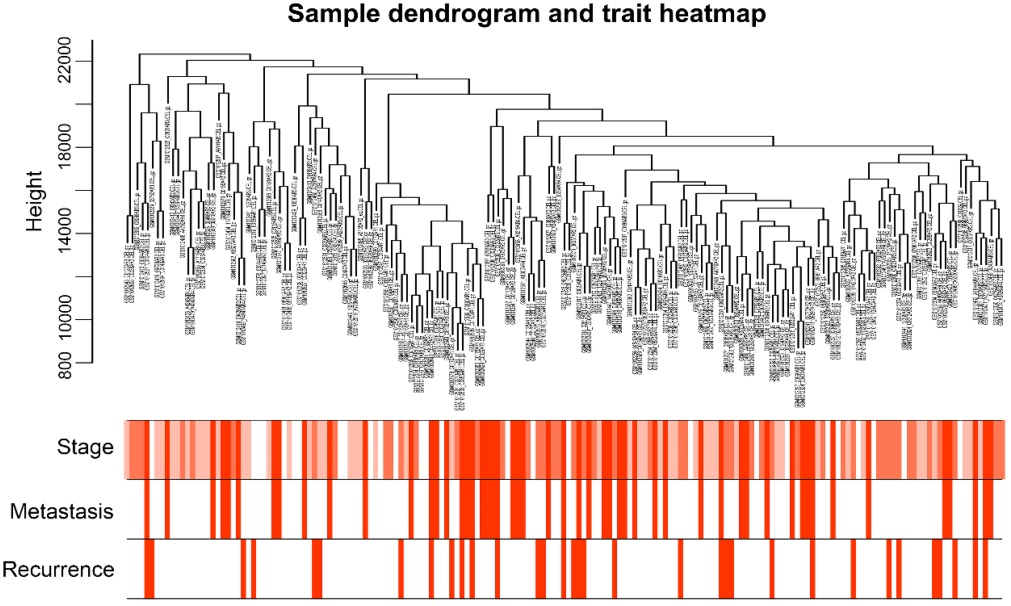
Sample dendrogram and the heatmap of trait indicators. The clustering was based on the expression data of GSE41258. The top 5,000 genes with the highest SD values were used for the analysis by WGCNA. The color intensity was proportional to tumor stage, metastasis and recurrence. WGCNA, weighted gene co-expression network analysis.

**Figure 3:**
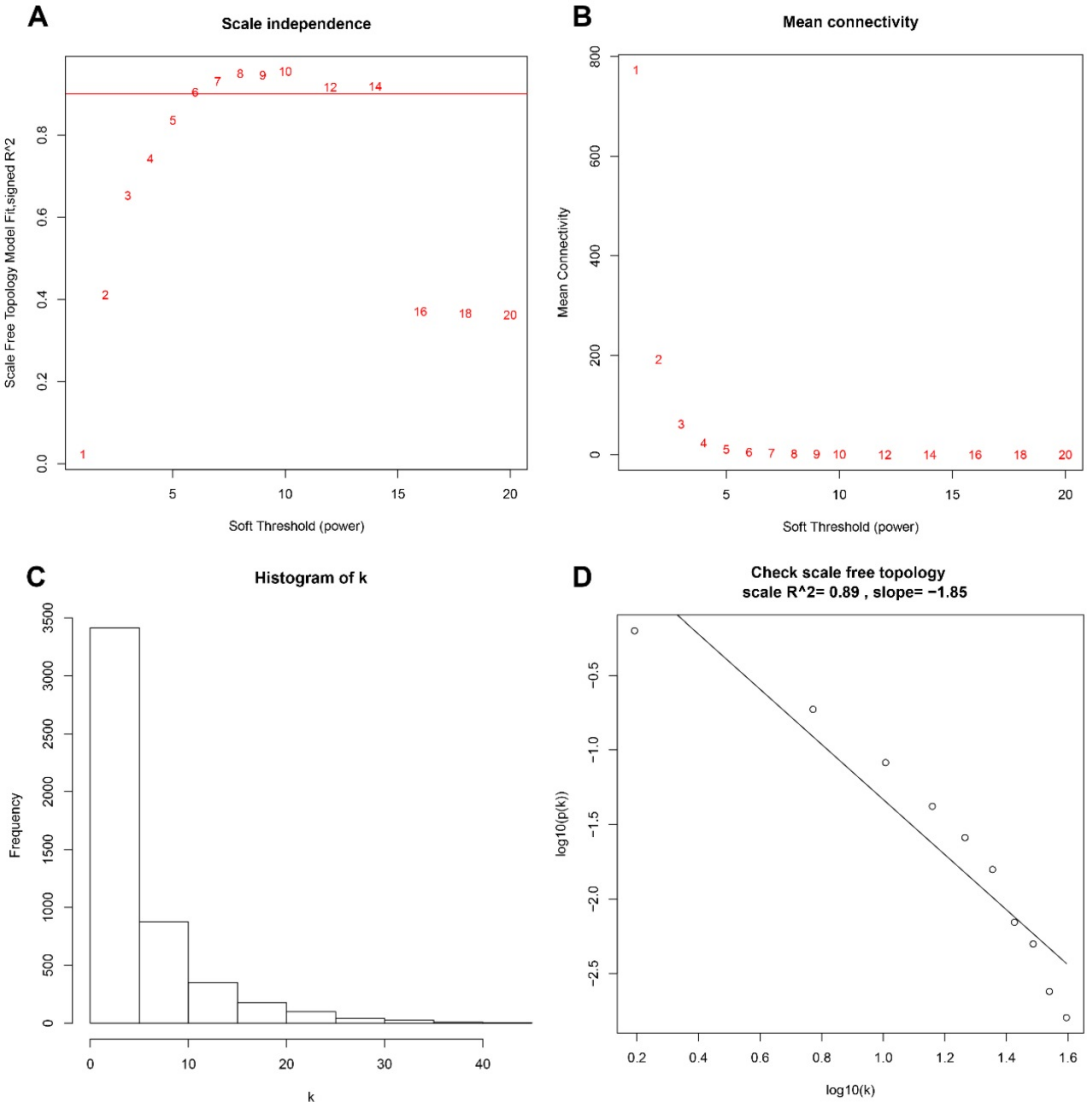
Determination of the soft‐thresholding power in the WGCNA. **a** Analysis of the scale-free fit index for various soft-thresholding powers (β). **b** Analysis of the mean connectivity for various soft-thresholding powers. **c** Histogram of connectivity distribution when β = 6. **d** Checking the scale free topology when β = 6. WGCNA, weighted gene co-expression network analysis.

**Figure 4:**
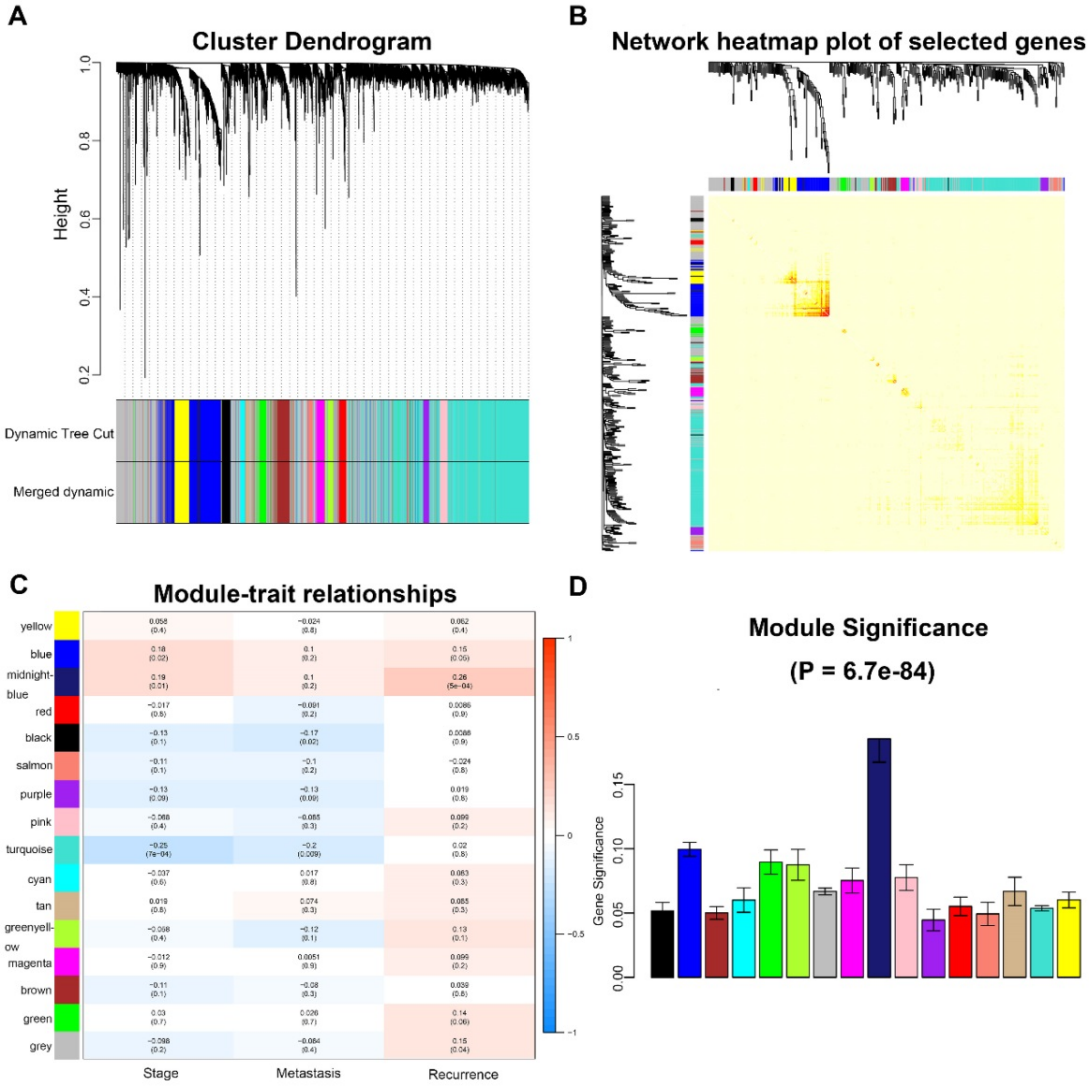
Identification of modules associated with the clinical traits of CRC. **a** Clustering dendrogram of genes based on a dissimilarity measure (1-TOM). **b** Topological overlap matrix plot. Genes in the rows and columns are sorted by the clustering tree. Different colors of horizontal axis and vertical axis represent different modules. The brightness of yellow in the middle represents the degree of connectivity of different modules. **c** Heatmap of the correlation between module eigengenes and clinical traits of CRC. **d** Distribution of average gene significance and errors in the modules associated with recurrence of CRC. CRC, colorectal cancer; TOM, topological overlap matrix.

A total of 173 qualified CRC samples with clinical data were included. Using the R package, “WGCNA”, genes which showed similar expression patterns were divided into different modules (Fig. 2). In our work, β = 6 (scale free R^2^ = 0.89) was screened as the soft-thresholding (Fig. 3). Then, we identified 16 modules and the network heatmap was shown (Fig. 4a, b). Relevance between key module and CRC recurrence was tested using two methods. Our results showed that the ME of the midnightblue module possessed the highest correlation with tumor recurrence ((P = 5×10^-4^, R^2^ = 0.26), Fig. 4c). Moreover, we also indicated that the MS of the midnightblue module was the highest among all modules (Fig. 4d), which was considered to have more connection with tumor recurrence. Therefore, we identified the midnightblue module to be a clinically significant module of interest in association with CRC recurrence in the training set.

### Hub gene identification

**Figure 5:**
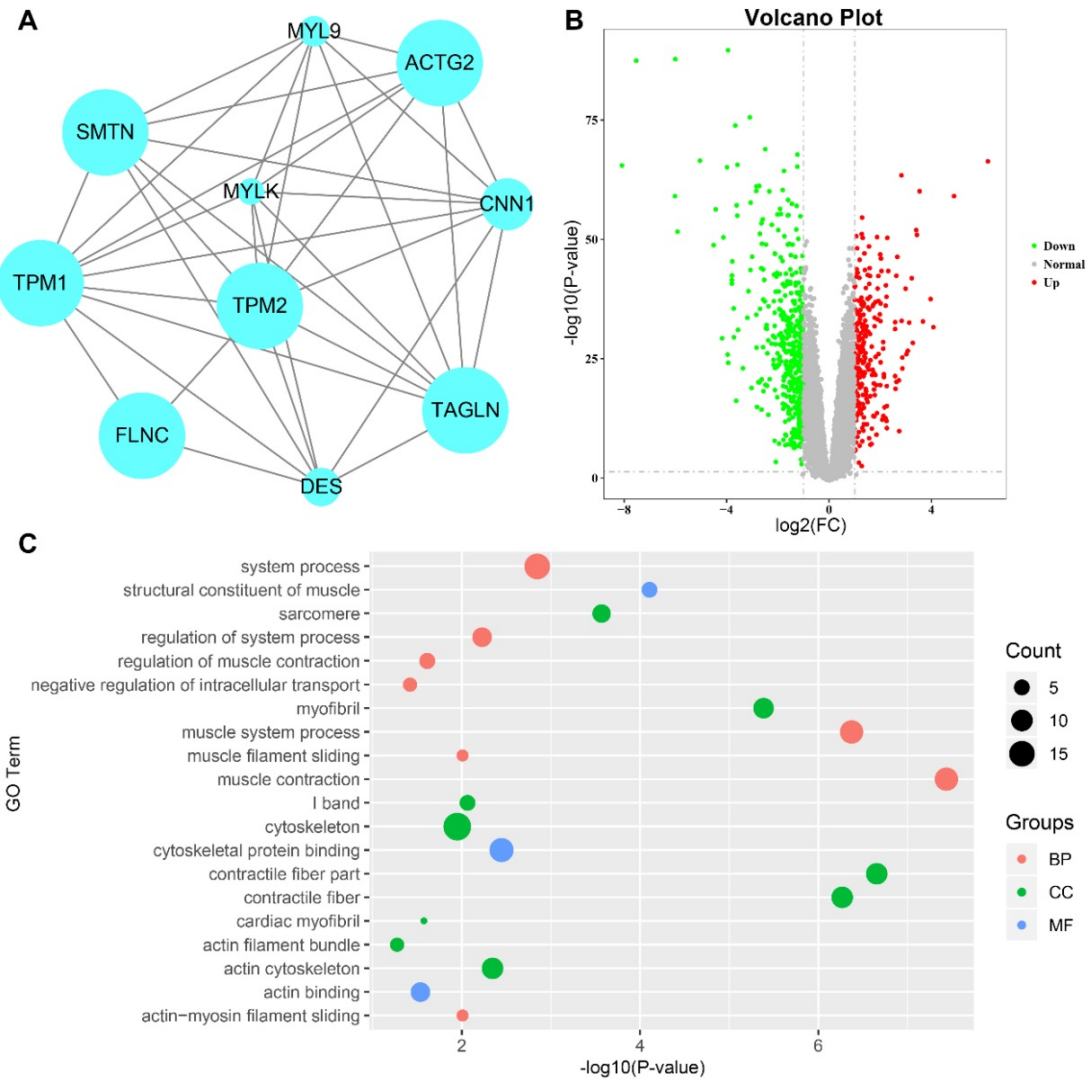
Identification of hub genes and GO analysis.** a** Protein-protein interaction network of the hub nodes. Nodes represent genes, and node size is correlated with connectivity of the gene by degree. **b** Volcano plot visualizing DEGs in GSE41258. The red dots represent all the up-regulated genes, the green dots represent all the down-regulated genes. **c** GO functional annotation genes in the midnightblue module. The x-axis represents the -log(P-value) of each term and the y-axis represents the GO terms. The size of the nodes is proportional to the number of genes. The red, green and blue color of the nodes represents biological process (BP), cellular component (CC) and molecular function (MF), respectively. GO, gene ontology; DEGs, differentially expressed genes.

In our study, 15 genes were identified to be hub genes, which had high connectivity in the midnightblue module (Table 1). In addition, all genes in the midnightblue module were uploaded to the STRING database (Fig. S5). According to the PPI network, four hub genes were considered as hub nodes (MYL9, MYLK, CNN1 and DES) as well, which were also DEGs in GSE41258 (Table 1, Fig. 5a, b). Therefore, MYL9, MYLK, CNN1 and DES were candidate hub genes. Furthermore, all genes in the midnightblue module were enriched for GO analysis. Our results showed top 20 GO terms and indicated that hub genes were significantly enriched in “muscle contraction”, “muscle system process”, “contractile fiber part”, “cytoskeleton” and “cytoskeletal protein binding” (P < 0.05, Fig. 5c).

### Validation of Hub Genes

**Figure 6:**
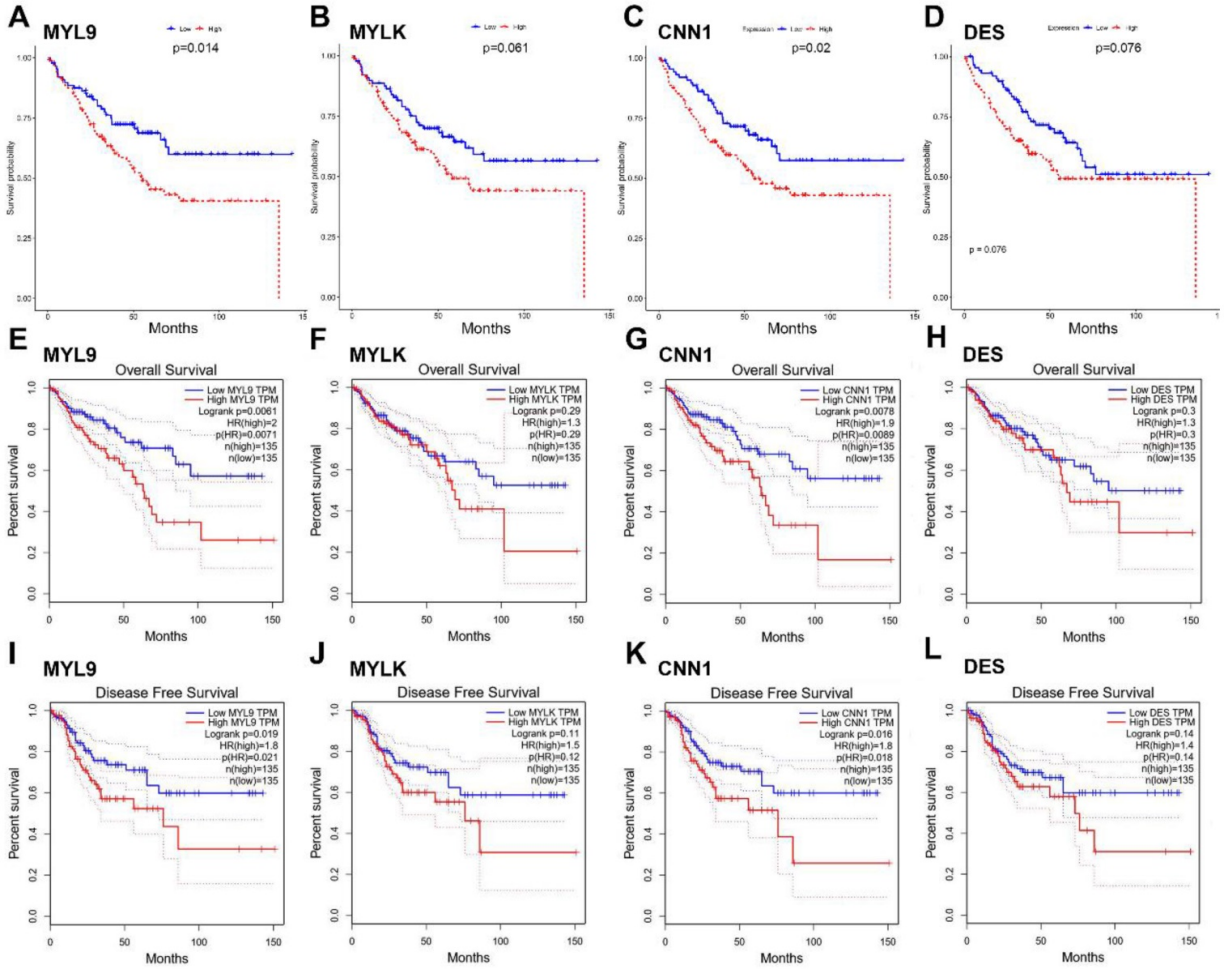
Survival analysis of hub genes. (**a**-**d**) Survival analysis of the association between MYL9, MYLK, CNN1 and DES expression and overall survival rate in CRC patients (based on the test set of GSE17536). (**e**-**h**) Kaplan-Meier survival curves obtained from the GEPIA database indicated that CRC patients with higher expression of MYL9 and CNN1 had a shorter overall survival time. (**i**-**l**) Kaplan-Meier survival curves obtained from the GEPIA database indicated that CRC patients with higher expression of MYL9 and CNN1 had a shorter disease-free survival time. CRC, colorectal cancer.

**Figure 7:**
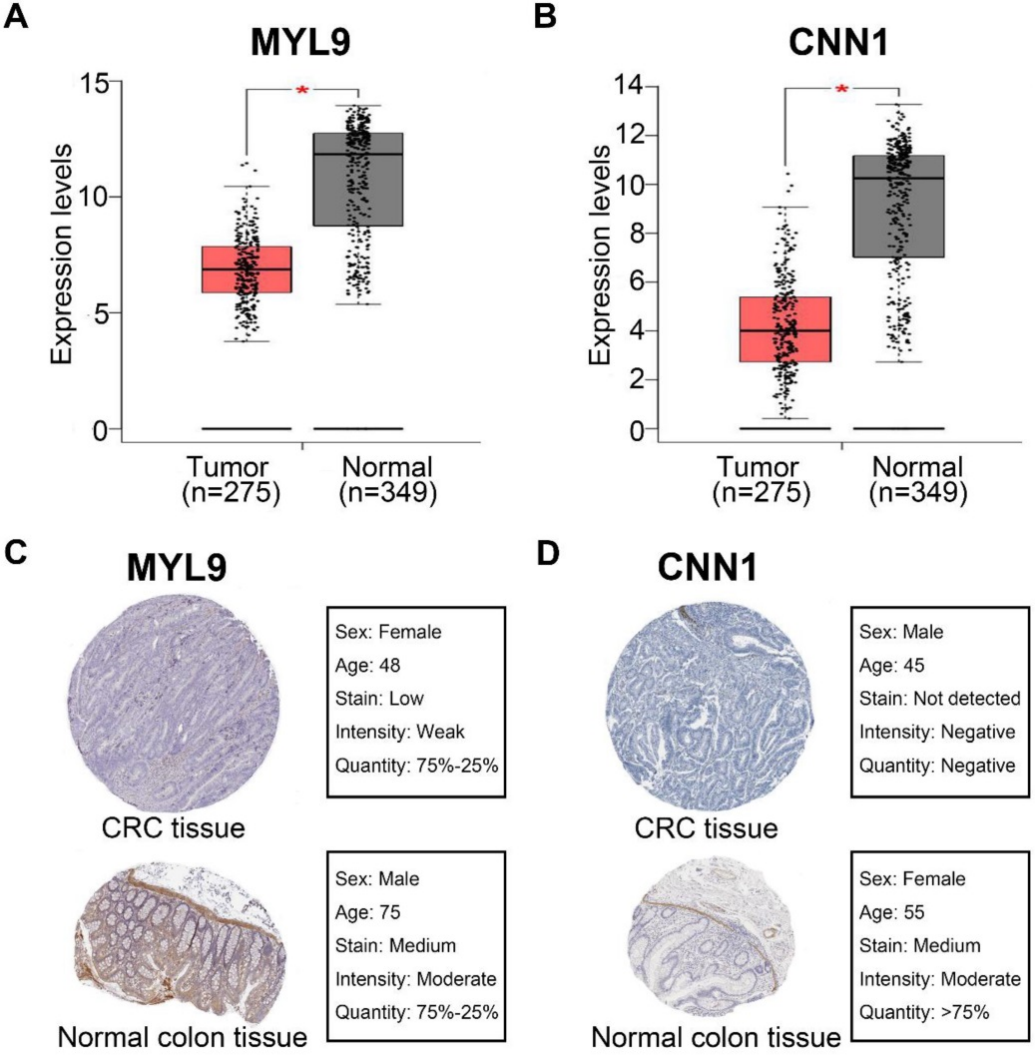
The expression levels of MYL9 and CNN1 in CRC. (**a**, **b**) GEPIA database showed lower mRNA expression levels of MYL9 and CNN1 in CRC tissues compared with normal colon tissues. (**c**, **d**) IHC analysis based on The Human Protein Atlas database indicated that the protein expression levels of MYL9 and CNN1 were lower in CRC tissues compared with normal colon tissues. CRC, colorectal cancer. IHC, immunohistochemistry.

The four candidate hub genes (MYL9, MYLK, CNN1 and DES) were chosen for validation. GSE17536 was used as a test dataset for overall survival analyses. Our results discovered that patients who had higher expression of MYL9 and CNN1 showed a significantly shorter overall survival time (MYL9 P=0.014; CNN1 P=0.02, Fig. 6a, c). More convincingly, GEPIA also indicated a decreased overall and disease-free survival rates in CRC patients with highly expressed (n=135) MYL9 and CNN1 (MYL9 overall: P=0.0071, disease-free: P=0.021; CNN1 overall: P=0.0089, disease-free: P=0.018, Fig. 6e-l). Moreover, GEPIA showed that mRNA expression levels of MYL9 and CNN1 in CRC tissues were both significantly lower than that in normal colon samples (P<0.05, Fig. 7a, b), which were consistent with results of DEGs analysis in GSE41258 dataset. In addition, IHC demonstrated that the protein expression levels of MYL9 and CNN1 were also downregulated in CRC tissues (Fig. 7c, d).

### Gene Set Enrichment Analysis

**Figure 8:**
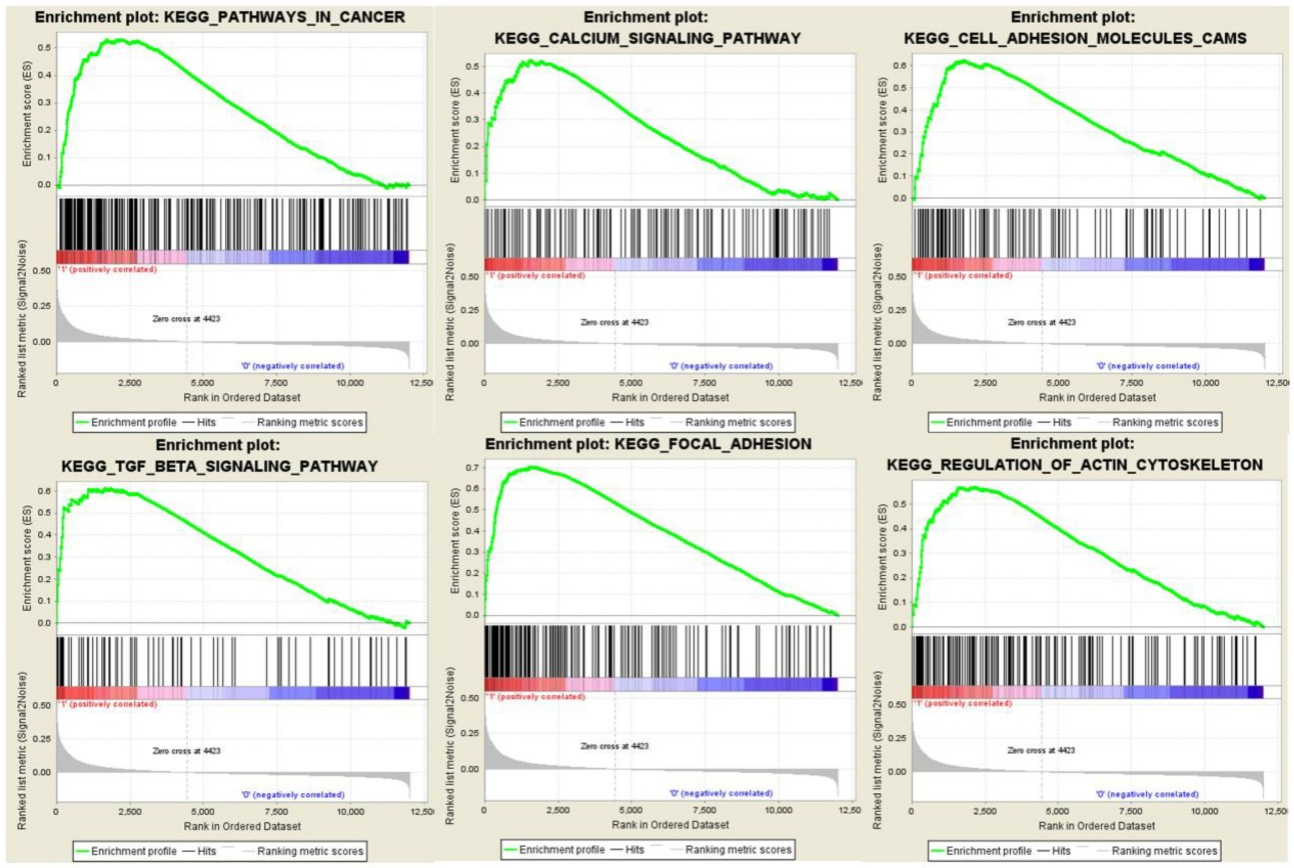
Gene set enrichment analysis. Six common and representative functional gene sets enriched in CRC samples with both highly expressed MYL9 and highly expressed CNN1 were listed. CRC, colorectal cancer.

To obtain further insight into the mechanisms of MYL9 and CNN1 in CRC, GSEA was performed to search KEGG pathways enriched in MYL9 and CNN1 highly expressed samples, respectively. Under the cut-off criteria FDR < 0.01, six common and representative functional gene sets were shown (Fig. 8), such as “pathways in cancer”, “calcium signaling pathway” and “focal adhesion”.

### qRT-PCR analysis

**Figure 9:**
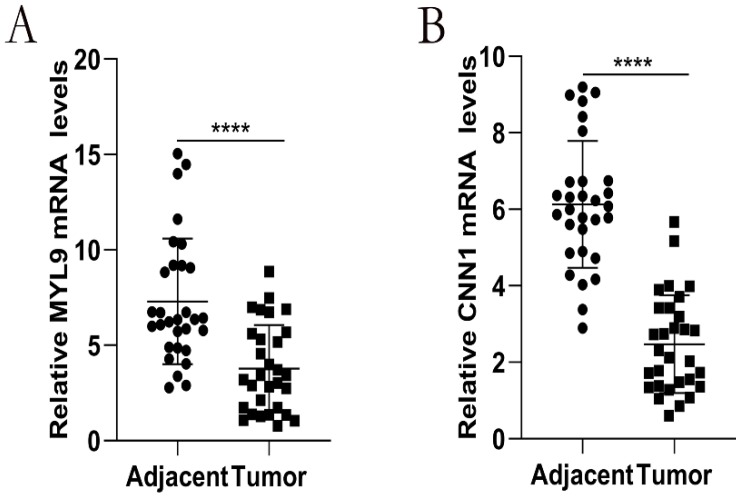
Relative expression levels of the hub genes validated by qRT-PCR analysis in 30 paired CRC and adjacent tissues. **a** The mRNA expression levels of MYL9 were down-regulated in CRC tissues.** b** The mRNA expression levels of CNN1 were down-regulated in CRC tissues. CRC, colorectal cancer.

We further performed qRT-PCR to validate the mRNA expression of MYL9 and CNN1 in 30 paired fresh CRC tissues and adjacent tissues. Our experimental results showed that MYL9 and CNN1 were highly expressed in adjacent tissues compared with CRC tissues (Fig. 9), which were consistent with results by microarray data.

## Discussion

In recent years, a systems biology approach, namely, WGCNA, has been widely used to identify potential and novel biomarkers in different kinds of tumors, such as adrenocortical cancer, clear cell renal cell cancer, oral squamous cell cancer and CRC 33-35, 13, 12. By performing WGCNA, Gao et al. reported distinct gene modules existing only in CRC liver metastatic tissues, which were not found in non-metastatic CRC samples 36. In addition, Liu et al. identified a novel prognostic maker, CENPA, which was associated with favorable survival outcome in CRC 14. As far as we know, numerous publications using WGCNA paid more attention to biomarkers for tumor stage of CRC. Although the TNM staging system was used to predict the recurrence of tumors 37, which was usually thought to be positively correlated with recurrence rate, however, it was contentious in a proportion of patients with CRC 38. Therefore, it is of great necessary to identify potential biomarkers which could not only predict the recurrence rate of CRC patients and make up for the deficiency of TNM staging system, but also have opportunities to identify high-risk patients at an earlier stage.

Until now, few studies have investigated potential biomarkers for CRC recurrence by performing WGCNA. In this work, WGCNA was applied in analysis of mRNA expression dataset GSE41258 in order to identify hub modules and genes associated with clinical features, such as tumor recurrence, which might be expected to be used as recurrence-free indicators in the future. Then, an independent validation dataset, GSE17536, was used to confirm our findings. Based on the clinical features, such as tumor stage, tumor metastasis and tumor recurrence, we finally chosen the tumor recurrence of CRC patients which was of interest to screen hub genes. The midnightblue module was identified, in which 15 genes were screened as hub genes; furthermore, four hub genes were also identified as hub nodes that showed a significant correlation with CRC recurrence. Among the four hub genes, MYL9 and CNN1 were found to be significantly correlated to overall survival as well as disease-free survival time of CRC patients. To investigate potential function and mechanisms related to hub genes, GO and GSEA analyses were performed. As a result, we found that MYL9 and CNN1 may participate in “pathways in cancer”, “calcium signaling pathway” and “focal adhesion”.

Myosin light chain 9 (MYL9), a protein encoding gene, is able to module the ATPase activity of myosin heads and regulate muscle contraction 39. It is well known that the phosphorylation of MYL9 plays a crucial role during the process of cell migration on solid substrates 40. Studies have detected the expression of MYL9 in various tumors, revealing that it was upregulated in breast cancer as well as liver cancer, while downregulated in prostate cancer and bladder cancer 41-44, however, it is controversial in CRC. Yan et al. reported that MYL9 was downregulated in CRC and lower expression level of MYL9 resulted in a decreased survival rate in CRC patients 45. Zhao et al. found that MYL9 was upregulated in the patients with early-onset CRC 46. In our study, we confirmed that MYL9 was downregulated in CRC samples and patients with low expression level of MYL9 had an increased survival time. Calponin 1 (CNN1) is one of the modulators of actomyosin contraction, which is also known to be involved in cancer development 47, 48. For instance, it has been reported that CNN1 expression could suppress ovarian cancer development 49. As for CRC, CNN1 was demonstrated to be expressed at a higher expression level in normal colon tissue compared to CRC samples 50, which was consistent with our results.

Generally speaking, almost 50% patients may suffer from tumor recurrence within the first year after initial resection, which may be closely correlated to the prognosis. To our knowledge, this is the first study to identify two potential biomarkers, MYL9 and CNN1, which were associated with CRC recurrence by using the WGCNA algorithm. Interestingly, we found that MYL9 and CNN1 were dramatically decreased in CRC samples compared with normal samples in both GSE41258 and GEPIA database, however, our survival analyses showed that higher expression of MYL9 and CNN1 correlated with poorer prognosis of CRC. We speculated that MYL9 and CNN1 may act as tumor suppressor genes in human bodies, however, with the development and progression of CRC, the two hub genes may be captured by tumor cells and turned to be harmful genes, therefore protecting tumor cells becomes the major role of MYL9 and CNN1. From another perspective, it is known that the stromal microenvironment in tumor tissue is different from the stroma of the corresponding normal tissue in many human cancers. Functionally, myosins are implicated in cell migration and adhesion, cells may exert force propelling the cell forward by contraction of actin cytoskeleton by activating of myosin II which is regulated by the phosphorylation of MYL9. As such, CNN1 is thought to play an essential role in organizing stable actin stress fibers. Additionally, GSEA found that the two hub genes were enriched in pathways in cancer. We therefore hypothesized that the phosphorylation of MYL9 or CNN1 is the key of cell migration process on solid substrates in tumor microenvironment but not in the normal stroma microenvironment, leading to the aggressive progression of CRC. Additionally, we also consider that the expressions of MYL9 and CNN1 are downregulated in CRC because of the general dedifferentiation of cancer cells, but those cancer cells with higher expression of MYL9 and CNN1 may be better suited for migration and metastasis compared with lower expressed ones. In this context, it is not surprising to observe that the two hub genes were decreased in CRC but negatively correlated to survival.

In conclusion, we identified two hub genes, MYL9 and CNN1, which were significantly related to the recurrence of CRC and may contribute to the improvement of recurrence-free survival time of CRC patients.

## Supplementary Material

Supplementary figures.

## Figures and Tables

**Table 1:** Hub genes in the midnightblue module related with CRC recurrence

Gene symbol	Probe	cor.geneModuleMembership	Hub gene in PPI network	DEG analysis	
				|logFC|	FDR
MYL9	201058_s_at	0.943041082	Yes	1.617677	1.03E-14
MYLK	202555_s_at	0.936476159	Yes	1.794758	7.82E-19
CNN1	203951_at	0.906218239	Yes	2.713818	1.34E-19
DES	202222_s_at	0.841739696	Yes	2.55989	7.76E-18
HSPB8	221667_s_at	0.913958361	No	2.087421	3.09E-27
SPARCL1	200795_at	0.900690649	No	1.531813	3.86E-21
KCNMB1	209948_at	0.890620367	No	1.567299	7.39E-26
KANK2	218418_s_at	0.880372051	No	1.084421	2E-20
AOC3	204894_s_at	0.880033125	No	1.91461	3.38E-27
CSRP1	200621_at	0.877492479	No	1.134281	1.7E-20
PPP1R12B	201957_at	0.872867041	No	1.795588	1.33E-33
PLN	204940_at	0.871769799	No	2.168066	4.12E-24
SYNM	212730_at	0.858816617	No	2.828553	1.48E-26
PDLIM3	209621_s_at	0.839725447	No	1.494531	3.86E-14
FERMT2	214212_x_at	0.808484526	No	0.804553	9.87E-12

Abbreviations: CRC, colorectal cancer; PPI, protein-protein interaction; DEG, differentially expressed gene; FC, fold change; FDR, false discovery rate.
