# Supplementary material for: Weighted gene co-expression network analysis identified MYL9 and CNN1 are associated with recurrence in colorectal cancer
Source: J Cancer. 2020 Feb 10;11(8):2348–59. doi: 10.7150/jca.39723 (PMC7052925; doi:10.7150/jca.39723)
Supplement: Supplementary file 1 — Supplementary figures. [file jcav11p2348s1.pdf]

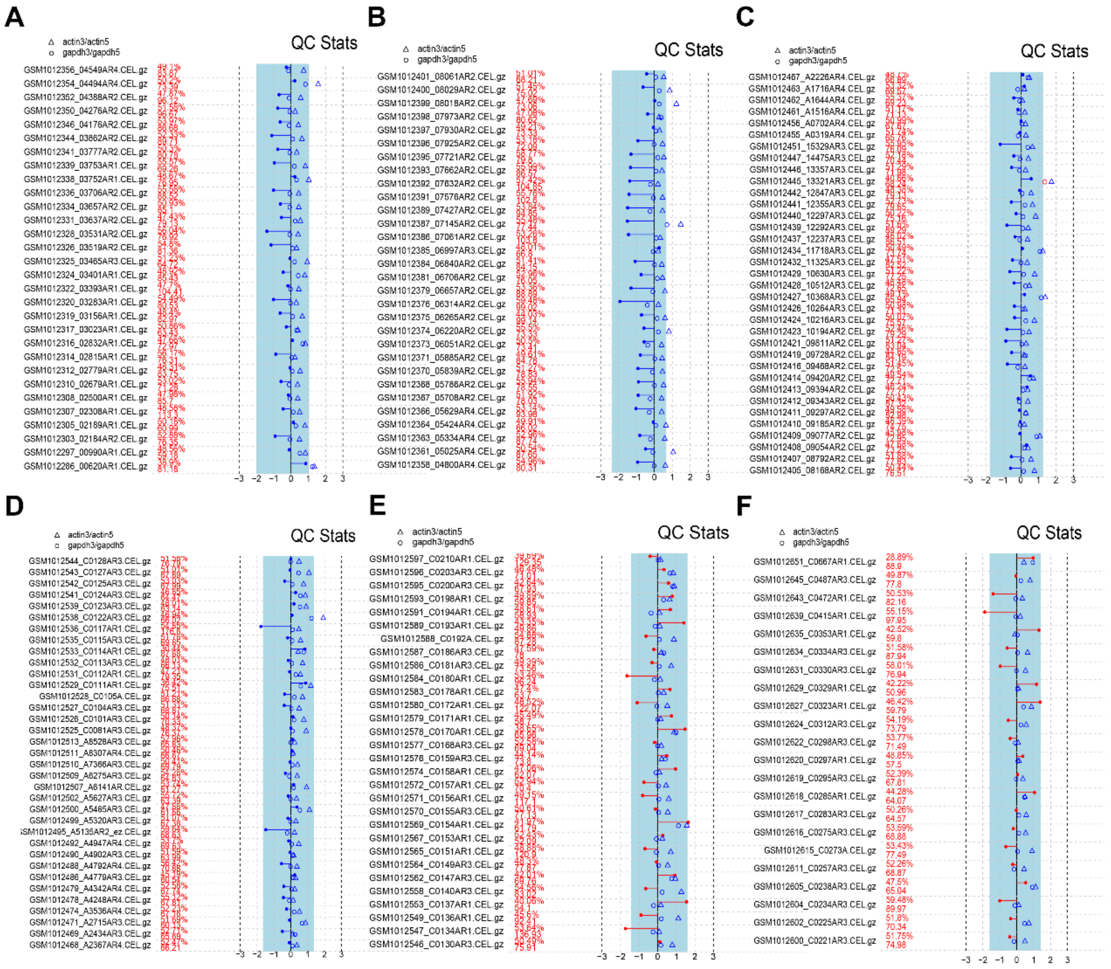

Fig. S1 An overview diagram of quality assessment of 182 CRC samples in GSE41258. CRC, colorectal cancer.

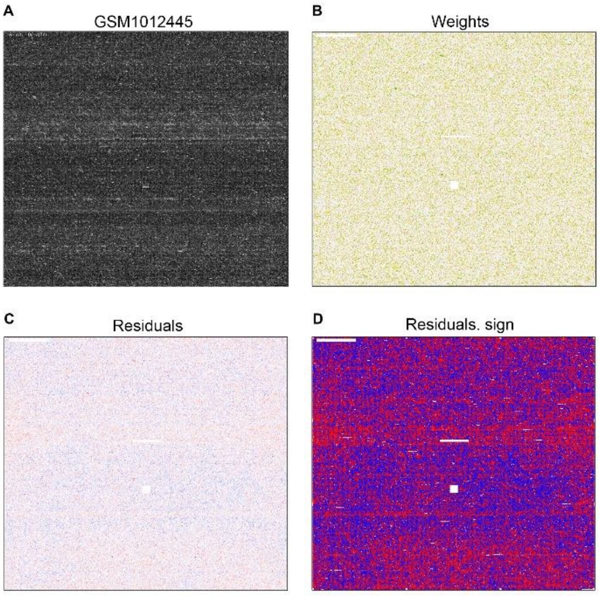

Fig. S2 Data fitting analysis of GSM1012445. **a** Grey-scale map. **b** Weights plot. (**c**, **d**) Residuals plots.

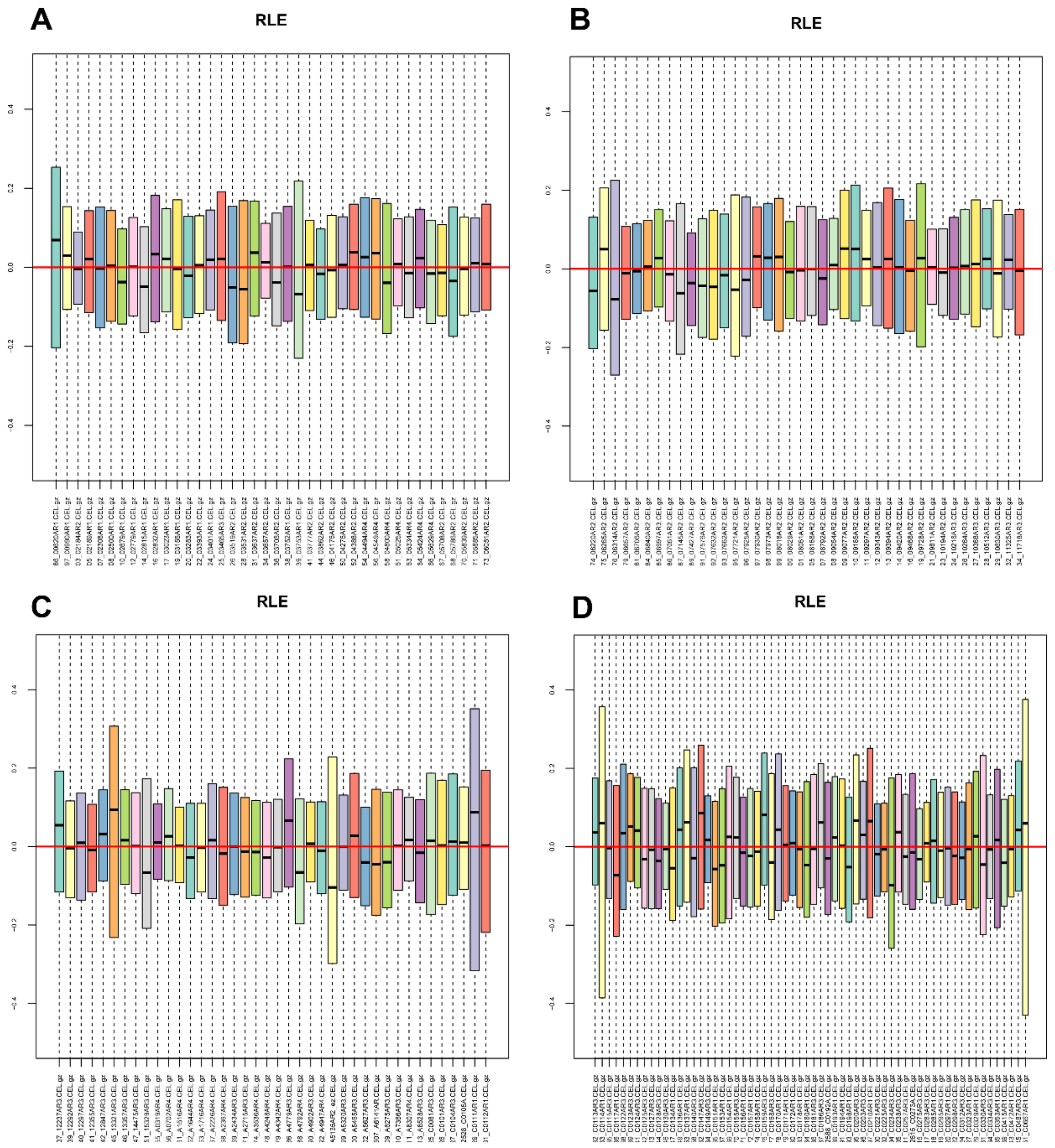

Fig. S3 The relative log expression (RLE) of 182 CRC samples in GSE41258. CRC, colorectal cancer.

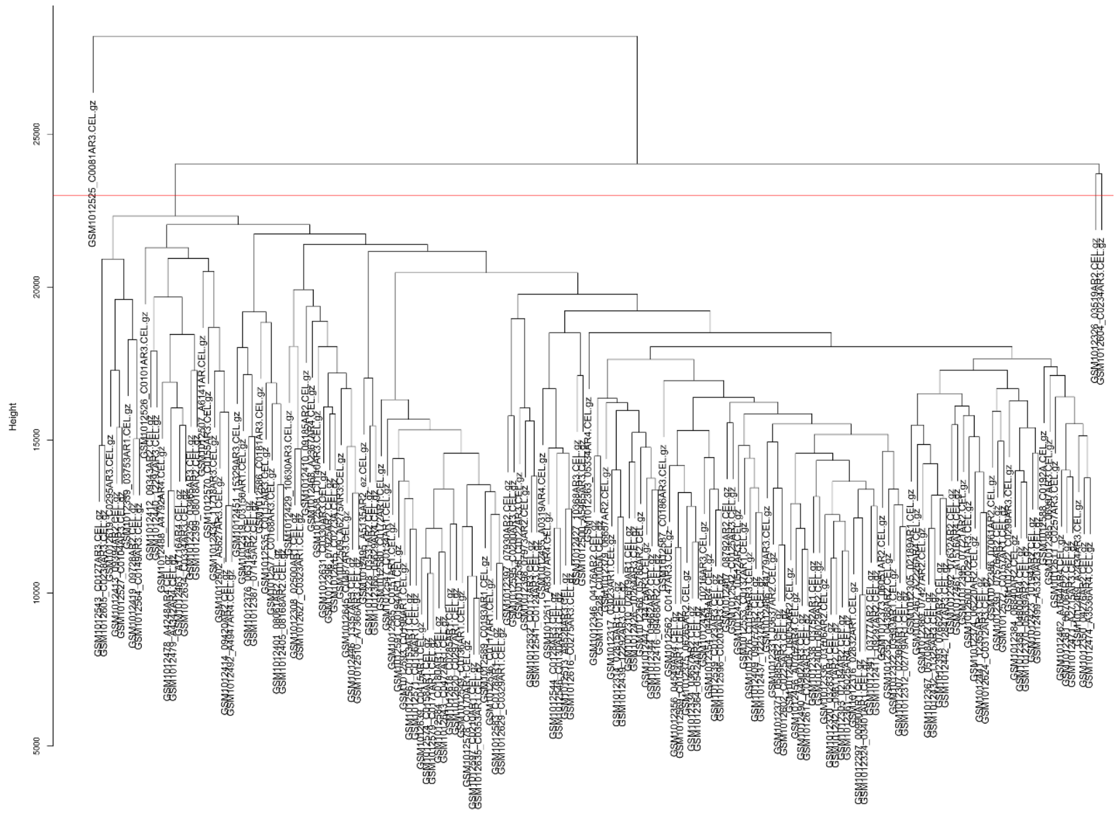

Fig. S4 Sample clustering to detect outliers in the training set of GSE41258.

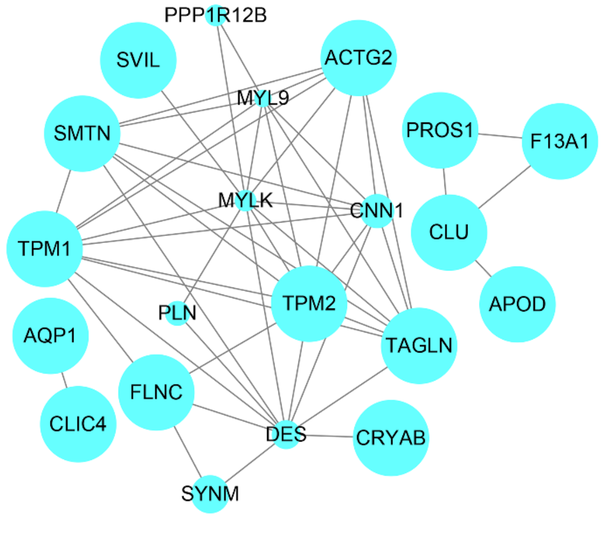

Fig. S5 Protein–protein interaction network of all genes in the midnightblue module. Nodes represent genes, and node size is correlated with connectivity of the gene by degree.
